# Clinicopathological Changes of the Maxillary Sinus in Patients With Ethmoidal Polyposis: A Prospective Observational Study

**DOI:** 10.7759/cureus.76730

**Published:** 2025-01-01

**Authors:** Dillip Kumar Samal, Amit Chirom, Saumyaranjan Mallick, Vikas Gupta, Ganakalyan Behera

**Affiliations:** 1 Otorhinolaryngology, All India Institute of Medical Sciences, Bhubaneswar, Bhubaneswar, IND; 2 Otorhinolaryngology, All India Institute of Medical Sciences, New Delhi, New Delhi, IND; 3 Pathology, All India Institute of Medical Sciences, New Delhi, New Delhi, IND; 4 Otorhinolaryngology, All India Institute of Medical Sciences, Bhopal, Bhopal, IND

**Keywords:** chronic sinusitis, ethmoid polyposis, maxillary sinus, nasal polypi, osteomeatal complex, paranasal sinus

## Abstract

Objective of study: To determine the clinical, radiological and histopathological changes in maxillary antrum compared to ethmoid sinuses in patients with chronic sinusitis with nasal polyposis.

Study design: This is a prospective observational study conducted in a tertiary care hospital.

Materials and methods: A total number of 30 primary cases of inflammatory nasal polypi, not responding to conservative measures, were evaluated clinically and radiologically. They underwent endoscopic sinus surgery, and all sinuses were cleared of diseases. Histopathological evaluation of both maxillary sinus and ethmoid sinuses was done and compared.

Results: Among 30 patients with 60 nasal cavities studied, nearly all showed partial or complete opacity of their maxillary antrum and blocked osteomeatal complexes on radiology. But, only a few patients (16.67%) showed frank polypi in their antrum intra-operatively. On histopathology, antral mucosa showed rare involvement, and the submucosa showed variable grades of inflammation, but it was significantly less compared to ethmoids.

Conclusion: Although maxillary antrum gets involved clinically in nearly all polyposis cases, pathological involvement is unlikely compared to ethmoid sinuses. Thus, it indicates a secondary inflammatory change in maxillary sinuses rather than primary involvement.

## Introduction

Nasal polyposis or ethmoid polyposis are very common entities affecting the middle-aged population. It potentially impairs quality of life more than perennial allergic rhinitis [1]. These are hyperplastic oedematous inflammatory mucosal outgrowth that arises de novo in the lateral wall of the nasal cavity at the uncinate process or bulla mucosa level, which releases inflammatory mediators [2]. As the inflammatory cascade involves both nasal and paranasal sinus mucosa, ‘rhinosinusitis’ is more appropriately used now than sinusitis [3]. Although polyposis can be unilateral in cases like antrochoanal polyp or allergic fungal sinusitis, bilaterality is the usual finding in the settings of chronic sinusitis with inflammatory nasal polyposis. The different proposed aetiologies are allergy, infection, inflammation, immunological reaction or genetic predispositions. Nasal polyposis can be seen in chronic rhinosinusitis, aspirin sensitivity, allergic fungal sinusitis, chronic granulomatous disease, Kartagener’s syndrome, and Churg-Strauss syndrome [4]. Initially, nasal polyposis involves the ethmoid sinuses and osteomeatal complex area. As the severity of the disease progresses, it involves the rest of the sinuses, causing mechanical airway obstruction with symptoms of nasal obstruction, discharge, post-nasal drip, sleep disorders, loss of smell, facial heaviness and even nasal deformity in rare cases [5]. Various authors have studied the histopathological characteristics of nasal polyposis and compared it with septal mucosa, middle turbinate and inferior turbinate mucosa but didn’t find any statistically significant difference between them, thus suggesting a generalised disease spectrum involving both nasal and paranasal sinus mucosa [6,7].

Ethmoidal polyposis originates from the middle meatus, with 97.4% originating from the anterior ethmoid complex followed by the uncinate process or infundibulum [8]. The maxillary sinuses and other sinuses may get involved later on either by the persistent disease process that causes ethmoidal polyposis or secondary to mechanical obstruction caused by a blocked osteomeatal unit (OMU). The persistence of symptoms, even with a patent maxillary ostium following endoscopic clearance of the OMU, suggests it to be a more generalised inflammatory pathology of sinuses rather than a simple mechanical obstruction. Thus, almost all patients need medical treatment with intra-nasal corticosteroid sprays post-operatively to control underlying inflammation and improve symptom scores. These hypotheses need further studies for a better understanding of the pathophysiology and management.

Our study aimed to examine the clinical and radiological findings in ethmoid and maxillary sinuses in cases of ethmoidal polyps and to compare further the histopathological changes in the mucosa and submucosa of these two subsites. Alternatively, nasal polyposis should be meant as ethmoidal polyposis for all discussion purposes.

## Materials and methods

This is a prospective observational study carried out in a tertiary care setup. The patients who were not responding to conventional medical treatment, including antiallergics, antibiotics and local steroidal nasal sprays, were included in this study. Patients with asthma, aspirin sensitivity, radiological evidence of allergic fungal rhinosinusitis, and significant medical co-morbidities were excluded from our study. Primary cases of ethmoidal polyposis patients without any prior surgical history were only included to avoid any disarray regarding their post-operative histopathology status in recurrence cases. Thirty otherwise healthy patients with chronic rhinosinusitis with nasal polyposis (CRSwNP with Type 2 inflammation) patients were included and studied over 18 months. Ethical approval was taken from the Institutional Ethics Committee, All India Institute of Medical Sciences, New Delhi (approval no. IESC/T-313).

The patients were evaluated regarding their related complaints and severity of nasal obstruction, nasal discharge, post-nasal drip, loss of smell, headache, facial heaviness or facial deformity. History was taken regarding the types of allergy, i.e., seasonal or perennial or associated allergic symptoms, if any. 

They underwent clinical examinations with office-based endoscopic evaluation to examine the presence and extent of polyposis, secretions, crusting and anatomical variations, if any. The extent of polyposis was evaluated and staged according to Johansson et al. in relation to middle meatus and inferiorly as follows: (i) Stage 0: No polyp; (ii) Stage 1: polyps restricted to middle meatus (mild polyposis); (iii) Stage 2: polyps extend beyond the middle meatus without going beyond the lower border of the inferior turbinate (moderate polyposis); (iv) Stage 3: polyps expand beyond the lower border of the inferior turbinate (severe polyposis) [9].

Radiological evaluation was done with high-resolution non-contrast computed tomography (non-contrast CT) in both coronal and axial sections to know the extent of disease involvement and anatomical variations, if any. The Lund-Mackay radiological staging system was followed for severity of involvement [10].

All 30 patients, i.e., 60 nasal cavities, underwent endoscopic sinus surgery under general anaesthesia. All surgeries were done by a single surgeon specialising in endoscopic sinus surgery. Intra-operatively, patients were examined and assessed for characteristics of the nasal mucosa, the character of discharge, the presence and extent of polyps, and the status of osteomeatal complexes. Uncinectomy was done, and the maxillary antrum was assessed for the presence of secretions, polypoidal changes or polyps. Secretions were cleared, and polyps and polypoidal mucosa debridement were done from the nasal cavity and involved sinuses using either a cold instrument or a microdebrider. Maxillary sinus mucosa was collected using antral forceps. The maxillary antral mucosa and polyps from ethmoid sinuses were fixed in 10% formalin and sent for histopathological examination. Patients were advised to use a saline nasal spray and repeated endoscopic nasal cleaning for two weeks. Then, they were started with nasal douching with budesonide nasal irrigation solution and were kept under close follow-up at monthly intervals for six months to see if any disease recurrences occurred. No significant complications were noted intra-operatively or in the immediate post-operative periods during their hospital stay.

The histopathological examination was carried out by the expert pathologist, who was blinded regarding the patient’s clinical, radiological and intra-operative findings. Specimens from ethmoids and maxillary sinuses were processed and embedded in paraffin. Serial 5µ thick sectioned specimens were stained with haematoxylin and eosin stains and were examined. The following characteristics were observed and graded semi-quantitatively: (i) Epithelium: 1. squamous metaplasia, 2. ulceration, 3. mucositis, 4. goblet cell reduction; (ii) Subepithelium: 1. basement membrane thickening, 2. submucosal oedema, 3. inflammatory cell infiltration (lymphocyte, eosinophil and plasma cell), 4. fibrosis and 5. submucosal congestion; (iii) Eosinophilic infiltrations were graded as 1: <20 per high power field, 2: 20-50 per high power field and 3: >50 per high power field; (iv) Goblet cell reduction was categorised as not reduced or reduced; (v) The remaining mucosal and submucosal characteristics were graded semi-quantitatively as 0: absent, 1: mild, 2: moderate and 3: severe. All cases were stained with Alcian blue-periodic acid Schiff (AB-PAS) and mucicarmine to characterise the nature of mucin.

Statistical data were analysed and were presented in frequency, mean, and median. The chi-square test was used to compare the characteristics between the ethmoids and maxillary sinuses, and a p-value <0.05 was taken as statistically significant.

## Results

A total of 30 patients with 60 pathological nasal cavities were intervened. Among them, 77% of patients were male patients, and 23% were female patients, with a mean age of 34.80 years (19 years to 50 years) and a standard deviation of 9.58. Nasal obstruction was the most common complaint in all patients, followed by nasal discharge (86.7% cases), decreased sense of smell (56.7% cases), post-nasal drip (53.3% cases) and headache (36.7% cases). 14 patients (46.7%) had a history of allergy in the form of nasal itching, irritation, excessive sneezing, and watering from the nostril and eye, whereas 16 patients (53.3%) had no history of any allergy. 

**Figure 1:**
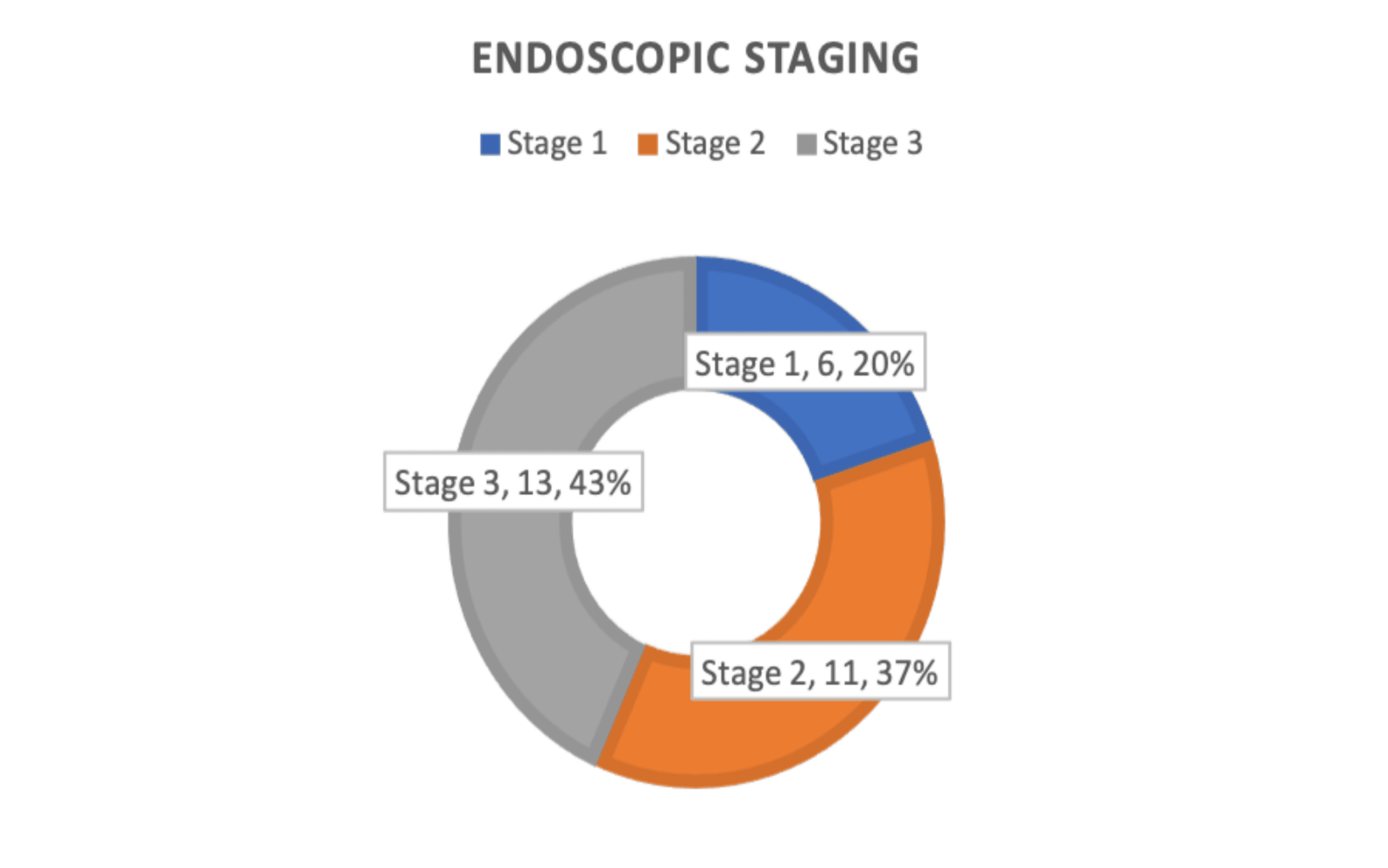
Pie chart showing endoscopic staging of patients at presentation.

Pre-operative endoscopic staging (Johansson et al.) [9] showed that 20% of patients had stage 1 disease, 37% of cases with stage 2 disease and 43% of cases with stage 3 disease (Figure 1).

**Figure 2:**
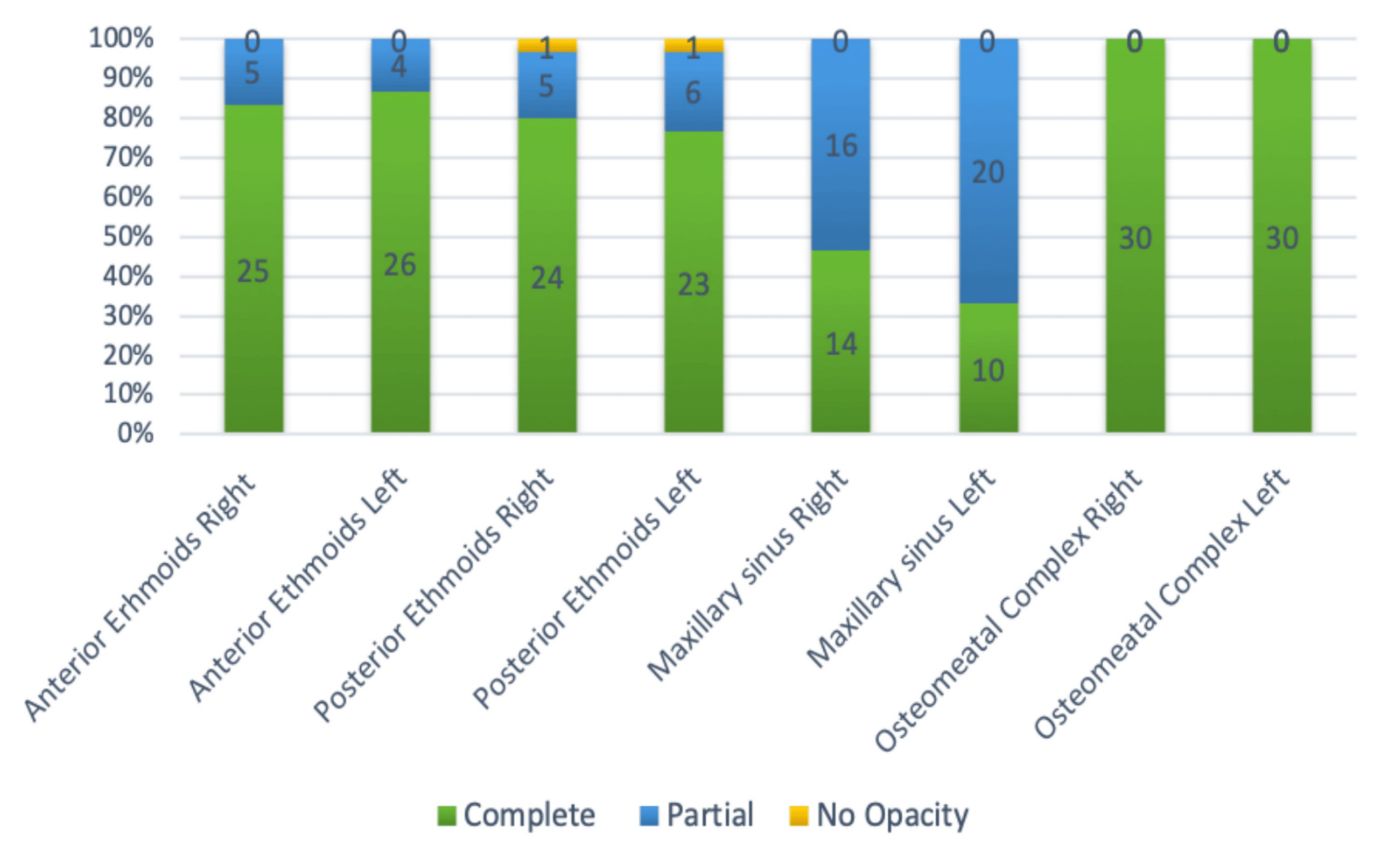
Bar diagram showing radiological scores of different sinuses: anterior ethmoids, posterior ethmoids, maxillary sinuses and osteomeatal units (using the Lund-Mackay scoring system).

On radiology, non-contrast CT scoring (Lund-Mackay staging) [10], more than 80 per cent of patients had a score of more than 15, and almost 57% of patients had a score of >20. The distributions of involvement of each sinus of both nasal cavities are represented in Figure 2.

**Figure 3:**
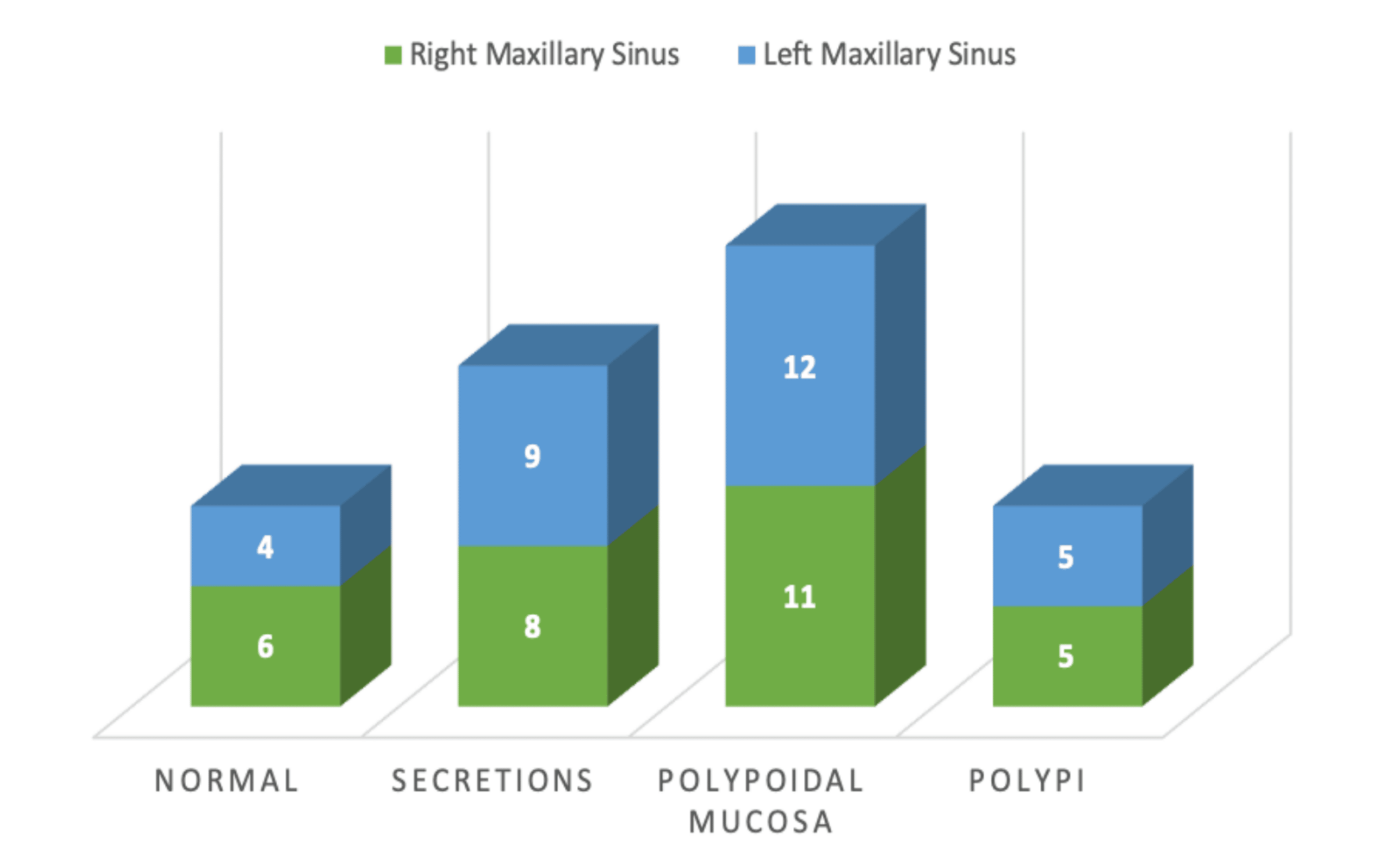
Bar diagram showing intra-operative findings of maxillary sinuses.

Intra-operative findings showed almost complete involvement of the anterior and posterior ethmoid sinuses occupied with multiple polyps. The osteomeatal complexes were blocked in all patients in both nasal cavities either by polypoidal mucosa or polyps. Out of 60 maxillary sinuses, 10 sinuses (16.6% cases) had normal mucosa, 17 sinuses (28.3% cases) had secretions, 23 sinuses (33.33% cases) had polypoidal mucosa and only 10 maxillary sinuses (16.67% cases) were filled with frank polyps. So, even at the advanced stage of the disease, maxillary sinus involvement was not so frequent (Figure 3).

On computer tomography, 56.7% of patients presented at a score >20 on Lund-Mackay staging (maximum score is 24). Anterior ethmoids in 51 sides (85%) showed complete opacity, and posterior ethmoids in 47 sides (78.34%) showed complete opacity, whereas in maxillary sinuses, 36 sides (60%) showed partial opacity, and only 24 sides (37.5%) showed complete opacity. The radiological correlation of severity of involvement between ethmoid sinuses (anterior and posterior) and maxillary sinuses was statistically significant, with a p-value < 0.05. The osteomeatal complexes bilaterally in all patients showed complete opacity radiologically.

On histopathological examinations, both ethmoidal polyps and maxillary antral mucosa specimens of 60 nasal cavities were stained and examined for the presence of mucosal ulceration, mucositis, goblet cell count, squamous metaplasia and basement membrane thickening. The submucosal layers were examined for inflammatory cell infiltration with lymphocytes, plasma cells or eosinophils, and any presence of congestion or fibrosis. Those characteristics in both specimens were analysed and graded semi-quantitatively as described, and the results were as presented here.

On examination of ethmoidal polyps, out of 30 patients with 60 specimens, 52 samples showed features of squamous metaplasia of their epithelium, 53 samples showed a reduction of their epithelial goblet cell count, 48 samples showed mucosal ulceration and 52 specimens had mucositis with lymphocyte infiltration in the mucosa. The basement membrane was thickened in 27 samples and submucosal fibrosis was seen in 30 samples. Submucosal congestion was seen in 15 samples and all specimens had submucosal oedema, although the severity was different. There were varying degrees of inflammatory cell infiltration present in polyps. Lymphocytic infiltration in the submucosa was seen in 57 of 60 nasal cavities. Eosinophilic infiltration was seen in all, but of varying degrees, graded as <20/HPF (high power filed) in 15 samples, 20 to 50 in 19 samples, and>50 counts in 26 samples. Plasma cell infiltration was significant and was seen in 45 specimens.

**Figure 4:**
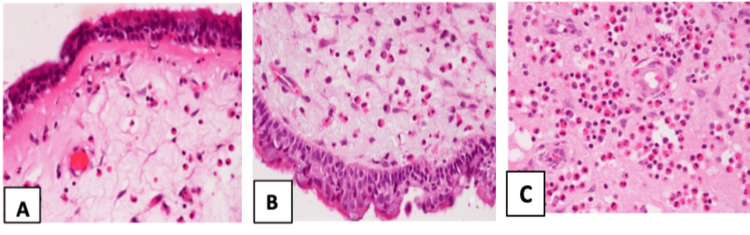
Submucosal eosinophilic infiltrations. (A) <20/high power field (HPF); (B) 20-50/HPF; (C) >50/HPF (haematoxylin and eosin staining on 400 X).

On examinations of maxillary antral mucosa, it showed a different histopathology compared to ethmoidal polyposis with a conspicuous absence of squamous metaplasia, mucosal ulceration, mucositis, goblet cell reduction, submucosal fibrosis and congestion. The basement membrane was thickened in only five samples, and submucosal oedema was observed in 23 of them. In their submucosa, lymphocyte infiltration was seen in 34 patients, and 29 patients showed plasma cell infiltration. Although 56 out of 60 samples showed eosinophilic infiltration, it was less severe with counts <20 in 35 samples, 20-50 range in 16 samples, and >50 in five samples only. A comparison of histopathological parameters showed a significant difference in the severity of changes in ethmoidal polyps vis-à-vis maxillary sinus mucosa (Table 1). The various grades of submucosal eosinophilic infiltrations have been depicted in Figure 4.

**Table 1:** Comparison of histopathological changes of ethmoidal polyposis and maxillary sinus mucosa.

Characters	Grade	Nasal polypi	Maxillary mucosa	P value
Squamous metaplasia	Absent	9	60	0.00001
	Mild	34	-	
	Moderate	12	-	
	Severe	5	-	
Goblet cell reduction	Normal	7	60	0.00001
	Reduced	53	-	
Mucosal ulceration	Absent	13	60	0.00001
	Mild	34	-	
	Moderate	10	-	
	Severe	3	-	
Mucositis	Absent	8	60	0.00001
	Mild	45	-	
	Moderate	5	-	
	Severe	2		
Submucosal fibrosis	Absent	29	60	0.00001
	Mild	20	-	
	Moderate	8	-	
	Severe	3	-	
Submucosal congestion	Absent	44	60	0.00001
	Mild	10	-	
	Moderate	4		
	Severe	2	-	
Basement membrane thickening	Absent	30	54	0.017
	Mild	24	5	
	Moderate	3	1	
	Severe	3	0	
Submucosal oedema	Absent	0	35	0.0001
	Mild	25	20	
	Moderate	26	5	
	Severe	9	0	
Lymphocyte infiltration	Absent	1	25	0.0086
	Mild	46	23	
	Moderate	5	6	
	Severe	8	6	
Plasma cell infiltration	Absent	16	30	0.0267
	Mild	32	21	
	Moderate	10	3	
	Severe	2	0	
Eosinophil infiltration	Absent	0	5	0.028
	<20	15	35	
	20-50	19	16	
	>50	26	4	

## Discussion

Nasal polyposis is a well-known, troublesome, benign disorder of the nose and paranasal sinuses affecting 1 to 4% of human populations [4]. But, the exact aetiology and pathogenesis remain unclear. Although it originates primarily in the middle meatus and ethmoid sinuses, later on, as the severity increases, it involves all the sinuses, including maxillary sinuses. It is a disease of the middle-aged population, 30-40 years being the most common age group, with a mean of 34.8 yrs in our study with a male predilection in a ratio of 3.28:1. These age groups are slightly younger in the Indian scenario than the populations described by Larsen and Tos as 51 years in male patients and 49 years in female patients as the mean age of involvement with a male predilection [11]. Lee et al. also described a mean age of 45.1 years [12]. Settipane et al. reviewed 211 patients and showed an equal distribution among male and female patients, i.e., 50.2% vs 49.8%, respectively [13]. We found nasal polyposis more commonly in non-allergic patients than allergic ones. A similar study was conducted by Settipane et al., which showed nasal polyposis was more common in non-allergic than allergic rhinitis and asthmatics patients [13]. On pre-operative endoscopic evaluation, it was found that most patients presented in their advanced stage of diseases with polyps filling the whole nasal cavity (stage 3).

Radiological findings showed significant differences in the involvement of ethmoid and maxillary sinuses. It more severely affects the ethmoid sinuses than the maxillary sinuses. Osteomeatal complexes of both sides showed complete opacity, which suggests the origin of the pathology, i.e., the complexes get blocked at the initial stage in almost all patients.

During the intra-operative evaluation, although almost all ethmoids were involved, the maxillary sinus showed partial involvement only, with 16.67% of sinuses showing frank polyps. So, compared with ethmoid sinuses, the severity of involvement of the maxillary sinuses was significantly less (p-value < 0.05).

On histopathological evaluation, nasal polyps showed variable grades of squamous metaplasia, mucositis, ulceration, basement membrane thickening, reduction of their goblet cell counts and submucosal inflammatory cell infiltrations. Pawankar has described four types of polyps based on their histology, i.e., eosinophilic oedematous type, chronic inflammatory or fibrotic type, seromucinous gland type and atypical stromal type, with the eosinophilic oedematous type being the common variant [4]. But, in our study, it couldn’t be characterised separately, and overlapping characteristics were noticed with variable grades of inflammatory cell infiltrations, with eosinophils being the commonest cell type. A Korean cohort has shown a preponderance of non-eosinophilic nasal polyposis than eosinophilic ones [14]. In their study of 30 patients, the non-eosinophilic type comprises 66.7% with a thinner basement membrane compared to the eosinophilic variant.

But, in our study, almost all cases showed lymphocytes and eosinophilic infiltrations with variable grades of plasma cell infiltrations. Most of the cases showed normal to mild thickened basement membrane. Maxillary antral mucosa in most cases was normal, suggesting that only in very few instances did antrum mucosa histopathologically get involved by polyps. The antral submucosa showed changes in basement membrane thickening, submucosal oedema and eosinophil-predominant inflammatory cell infiltrations. However, the involvement severity was less than in ethmoidal polyps, and there was a significant difference among them with a p-value < 0.05. The eosinophilic infiltrations in nasal polyps and maxillary antral mucosa are directed towards a more intrinsic cause affecting the whole mucosa. However, these findings should be compared with normal antral mucosa taken as control before commenting on underlying aetiology.

 In all cases, post-operative histopathology was only confirmed as chronic inflammatory nasal polyps. This correlates well with a study conducted by Yaman et al., who studied 85 patients with bilateral nasal polyps and no specimens presented any occult pathology [15]. 

This is a single-centre, interventional study with a relatively small sample size. The molecular and immunopathological study of nasal polyps is not included in this study. These are the limitations of our study.

## Conclusions

From radiological, intra-operative and histopathological evaluations, it was concluded that maxillary antrum was less common and less severely involved in cases of ethmoidal polyposis. However, it showed variable grades of inflammations with eosinophilic infiltrations like that of nasal polyps. Thus, although maxillary sinus involvement is a manifestation of the underlying disease spectrum, as previously thought, the severity increases after the blockade of the osteomeatal complexes.

In patients with ethmoid polyposis, the radiological involvement of the maxillary sinus doesn’t correlate well with the histopathological changes. Thus, conservative functional ethmoidectomy should be preferred for the management of inflammatory nasal polyposis rather than a radical approach.
